# Supplementary material for: MetaQTL: a package of new computational methods for the meta-analysis of QTL mapping experiments
Source: BMC Bioinformatics. 2007 Feb 8;8:49. doi: 10.1186/1471-2105-8-49 (PMC1808479; doi:10.1186/1471-2105-8-49)
Supplement: Additional File 7 — MetaQTL Package : jar file and tutorial. This Zip archive contains both the MetaQTL JAR file and the files of the tutorial. [file 1471-2105-8-49-S7.zip › org.inra.metaqtl/doc/org/metaqtl/MetaDico.html]

MetaDico


|  |  |  |
| --- | --- | --- |
| **Overview** \| **Package** \| **Class** \| **Use** \| **Tree** \| **Deprecated** \| **Index** \| **Help** | |  |
| **PREV CLASS**   **NEXT CLASS** | **FRAMES**    **NO FRAMES**     **All Classes** |  |
| SUMMARY: NESTED \| FIELD \| CONSTR \| METHOD | DETAIL: FIELD \| CONSTR \| METHOD |  |


---


## org.metaqtl Class MetaDico

```
java.lang.Object
  org.metaqtl.MetaDico
```

---

``` public class MetaDico extends java.lang.Object ```

This class represents a dictionnary of synonyms. In this context the entries
of the dictionary are standard names and the definition for each entry is just
a list of other terms which are synonyms.

---

**Constructor Summary**

`MetaDico()`


| **Method Summary** | |
| --- | --- |
| `void` | `addTerm(java.lang.String term, java.lang.String syn)` |
| `java.util.List` | `getSynonymous(java.lang.String name)` |
| `java.lang.String` | `getTerm(java.lang.String name)` |
| `java.util.Enumeration` | `getTerms()` |
| `void` | `setTerm(java.lang.String name, java.util.List synonymous)` |

**Methods inherited from class java.lang.Object**

`clone, equals, finalize, getClass, hashCode, notify, notifyAll, toString, wait, wait, wait`

**Constructor Detail**

### MetaDico

```
public MetaDico()
```


**Method Detail**

### getTerm

```
public java.lang.String getTerm(java.lang.String name)
```

:   **Parameters:**: `name` - **Returns:**

---


### getSynonymous

```
public java.util.List getSynonymous(java.lang.String name)
```

:   **Parameters:**: `name` - **Returns:**

---


### getTerms

```
public java.util.Enumeration getTerms()
```

:   **Returns:**

---


### setTerm

```
public void setTerm(java.lang.String name,
                    java.util.List synonymous)
```

:   **Parameters:**: `name` -: `synonymous` -

---


### addTerm

```
public void addTerm(java.lang.String term,
                    java.lang.String syn)
```


---


|  |  |  |
| --- | --- | --- |
| **Overview** \| **Package** \| **Class** \| **Use** \| **Tree** \| **Deprecated** \| **Index** \| **Help** | |  |
| **PREV CLASS**   **NEXT CLASS** | **FRAMES**    **NO FRAMES**     **All Classes** |  |
| SUMMARY: NESTED \| FIELD \| CONSTR \| METHOD | DETAIL: FIELD \| CONSTR \| METHOD |  |


---
